# Patients with suspected pulmonary hypertension based on echocardiography in Behçet's disease: a 5-year follow-up study

**DOI:** 10.1186/s13023-025-03825-x

**Published:** 2025-06-11

**Authors:** Mustafa Ekici, Alper Sarı, Berkan Armağan, Erdinç Ünaldı, Büşra Fırlatan, Gözde Sevgi Kart Bayram, Buğu Bulat, Uğur Nadir Karakulak, Levent Kılıç, Barış Kaya, Ömer Karadağ, Ali Akdoğan

**Affiliations:** 1 Department of Rheumatology, Faculty of Medicine, Hacettepe University, Altındağ, Ankara Turkey; 2 Department of Rheumatology, Etlik City Hospital, Yenimahalle, Ankara Turkey; 3 Department of Rheumatology, Bilkent City Hospital, Çankaya, Ankara Turkey; 4 Department of Cardiology, Faculty of Medicine, Hacettepe University, Altındağ, Ankara Turkey

**Keywords:** Behçet's disease, Pulmonary hypertension, Transthoracic echocardiography, Pulmonary endarterectomy

## Abstract

**Background:**

This study investigates the long-term functional and transthoracic echocardiographic (TTE) progression in Behcet’s disease (BD) patients with pulmonary hypertension (PH), aiming to address knowledge gaps regarding PH in BD.

**Methods:**

This study included 17 BD patients with PH detected by TTE and 6 patients with pulmonary artery involvement but without PH from a previous study at Hacettepe University. PH was defined as an estimated systolic pulmonary artery pressure (sPAP) ≥ 40 mmHg. TTE was conducted by a cardiologist blinded to clinical information, with sPAP calculated using the simplified Bernoulli equation. Right heart catheterization was performed only in two patients who underwent pulmonary endarterectomy operations.

**Results:**

All 23 patients were reached by telephone, and 13 of the 17 with PH attended the clinic for reevaluation and a TTE was performed. After 5 years, all patients were alive. Clinical worsening or FC progression was not observed in any patient with PH except the ones with Group IV PH. Pulmonary endarterectomy (PEA) was performed in 2 of the 4 Group IV PH patients during follow-up. Additional Group II PH diagnosed due to mitral valve disease in a patient with Group IV PH. Other than the ones who underwent PEA there were no increase in sPAP of the BD patients with PH. Among patients with PAI but without PH during first evaluation; only one was mildly symptomatic after 5 years who had a normal sPAP.

**Conclusion:**

Progressive PH was observed only in Group IV PH patients, while other groups remained generally asymptomatic. Although the number of patients enrolled in this study is limited implementation of TEE to the follow-up of BD patients with PAI for screening and monitoring PH can be beneficial for early diagnosis and defining treatment strategy.

## Introduction

Behçet's disease (BD) is a systemic vasculitis with unknown cause, affecting blood vessels of varying types and sizes. It's characterized by recurrent oral, genital, and intestinal ulcers and skin lesions. Other manifestations include arthritis, uveitis, central nervous system (CNS) involvement, venous-arterial thrombosis, and arterial aneurysms [1, 2].

Pulmonary hypertension (PH) is defined as a mean pulmonary artery pressure (mPAP) of more than 20 mmHg during a resting right heart catheterization (RHC). A notable rise in the risk of mortality and hospitalization becomes apparent when the mean pulmonary arterial pressure (mPAP) exceeds 20 mm Hg [3]. The clinical classification of PH includes 5 groups: pulmonary arterial hypertension (group I), PH associated with left heart disease (group II), PH associated with lung diseases and/or hypoxia (grup III), PH associated with pulmonary artery obstructions (group IV), PH with unclear and/or multifactorial mechanisms (group V) [4, 5]. Transthoracic echocardiography (TTE) is a recommended and widely used method for screening pulmonary hypertension, with a systolic pulmonary arterial pressure (sPAP) measurement of ≥ 40 mmHg obtained via TTE indicating the potential existence of PH [4–6].

The incidence of vascular events in patients with BD is estimated to be between 15 and 50% [1]. The predominant pulmonary vascular manifestations of BD are pulmonary artery aneurysm (PAA) and pulmonary artery thrombosis (PAT). PH may potentially complicate and worsen the prognosis of pulmonary arterial involvement in patients with BD [7]. In addition, all other groups of PH can also be seen the course of BD [6]. In Behçet's disease, vascular involvement is characterized by immune-mediated inflammation, leading to vascular wall damage and thrombosis. Obstructions in the pulmonary circulation are considered the primary cause of PH in patients with PAI. Additionally, vasculitis of small pulmonary arteries, systemic-to-pulmonary shunts (fistulas), and compression of pulmonary arteries by aortic aneurysms can also contribute to increased pulmonary artery pressure [8–10]. Previous studies reported a prevalence of PH in BD ranging from 9 to 65% based on TTE [6, 7, 11]. Recently, in our study using TTE, the overall PH frequency was 11% in patients with BD, whereas PH frequency was 40% in patients with PAI [6]. In literature, there is a lack of comprehensive data regarding the long-term prognosis and subsequent monitoring of sPAP in BD patients with PH. The aim of this study is to re-evaluate the clinical status (mortality, functional capacity, change in sPAP measurements by TTE) of BD patients with PH after 5 years of follow-up.

## Methods

### Patients

This study included 17 patients with BD in whom PH was detected by echocardiography in a study conducted at Hacettepe University rheumatology clinic 5 years ago [6]. In addition, 6 patients with pulmonary artery involvement but without PH from the previous study were also included. All included patients met the International Study Group Criteria for diagnosis of BD [12]. This study included patients with TTE-determined PH ≥ 40 mmHg from a previous study.

### Study design

An attempt was made to reach all patients by phone, and patients with PH detected in the first study were invited to the clinic for re-evaluation. A comprehensive medical history was obtained through telephone interviews from patients with PAI but without PH and patients with PH who were unable to attend the clinic. Medical history included demographics, World Health Organization functional class (WHO-FC) [13], new onset cardiac/pulmonary disease and cardiopulmonary surgery. Physical examination of all the patients who attended to clinic were performed and they were also evaluated with TTE by the same cardiologist (UNK) who was blinded to patient's clinical information. Transthoracic echocardiography was performed with GE VIVID E9 (MN, USA) by 2.5–3.5 MHz transducer. Systolic PAP was calculated by the Bernoulli equation from the peak tricuspid regurgitation jet velocity (V) by adding the estimated right atrial (RA) pressure (sPAP = 4(V)2 + RA pressure). If a high quality jet flow could not be obtained, the contrast agent and/or leg elevation were used. According to the current ESC Pulmonary Hypertension guideline, in addition to the TR jet velocity, pulmonary arterial dimension on parasternal short axis view, basal right ventricular dimension, inferior vena cava dimension on subcostal window, right atrial area on apical four chamber, tricuspid annular plane systolic excursion through M-Mode function, and the presence of pericadial effusion were also recorded and considered in terms of PH diagnosis [5]. BD patients were classified into PH groups according to the probable causes of PH, in line with current guidelines, through a multidisciplinary evaluation [6]. The study protocol was approved by Hacettepe University non-interventional studies ethics committee with the number GO 23/184.

### Statistical analysis

Statistical analysis was performed using the IBM SPSS Statistics version 24.0 (IBM Corp., Armonk, NY, USA). Descriptive statistics were expressed as mean (standard deviations [SD]) for normally distributed and as median (interquartile range, IQR) for non-normally distributed data.

## Results

All of 23 patients were reached by telephone (17 patients with PH and 6 patients with PAI but without PH). 13 of 17 patients attented to clinic for reevaluation. 12 of 17 patients with PH were male, the median age was 52 years (40–66) and the median disease duration was 20 (12.5–34) years. Four of 6 patients with PAI but without PH were male, their median age was 49.1 years (42.5–70.1), and median disease duration was 12.1 (10.3–34.9) years.

In the first study, PH groups of patients determined by multidisciplinary approach were as follows; Group II in 9 patients (one had both group II and V PH), Group III in 1 patient and Group IV in 4 patients. The PH group could not be determined in 3 patients. All patients were alive after 5 years and none of the patient worsened or FC progressed except the ones with Group IV PH. Additional Group II PH diagnosed due to mitral valve disease in a patient with Group IV PH during this time period. Another patients had new gastrointestinal system involvement and treated with salazopyrin. Demographic and the clinical characteristics of patients with PH at year 5 follow-up are shown in Table 1.

**Table 1:** Clinical characteristics of Behçet's patients with pulmonary hypertension after five years

Patient number	Age/Sex	BD involvement	Disease duration (year)	PH class**	First NYHA FC	Last NYHA FC	First sPAP	Last sPAP	BNP (pg/ml)	Additional new and/or progressive cardiopulmonary diseases***	Treatment at first TTE evaluation	Treatment at last TTE evaluation ****
1	73, Female	Vascular Neurological	46	II	II	II	50 mmHg	50 mmHg	55	No change	Colchicine, warfarin	Colchicine, warfarin
2	59, Female	Vascular Ocular	32	IV	I – II	II	50 mmHg	45 mmHg	380	Endarterectomy MVR	Colchicine, IFN, warfarin, BP	Warfarin, Prednisolone
3	54, Male	Vascular	22	IV	III	III	50 mmHg	40 mmHg	61.6	Endarterectomy on riociquat	Colchicine, prednisolone, MMF, warfarin, diltiazem	Prednisolone, warfarin, SLZ (for GIS involvement), diltiazem, riociquat
4	62, Male	Ocular Neurological	33	II	I – II	I	45 mmHg		30.3	No change	Colchicine, IFN	Colchicine, azathioprine
5	46, Male	Ocular	18		I	I	45 mmHg			No change	Colchicine, azathioprine, prednisolone	Colchicine
6	70, Male	Vascular Ocular	43	III	II	I	45 mmHg	35 mmHg	33.7	No change	Colchicine, azathioprine, BP	Colchicine, azathioprine
7	76, Female	Ocular	51	II	II	I	45 mmHg	40 mmHg	329.1	No change	Colchicine	Colchicine
8	38, Male	Vascular	10	IV	II	II	45 mmHg	35 mmHg	10	No change	Prednisolone, interferon-alpha, colchicine, warfarin	MMF, enoxaparin ^+^
9	59, Male	Ocular	13	II	I	I	40 mmHg	35 mmHg	10	No change	Colchicine, BP	Colchicine, BP
10	45, Male	Vascular Ocular	20	IV	I	I	40 mmHg	30 mmHg	11.9	No change	Prednisolone, azathioprine	Azathioprine
11	51, Male	Ocular GIS	22	II	I	I	40 mmHg	10 mmHg	17.7	No change	Colchicine, SLZ	Colchicine, SLZ
12	42, Male	Vascular	8	II	I	I	40 mmHg	20 mmHg	12.3	No change	Azathioprine, colchicine, warfarin, prednisolone	Azathioprine, colchicine
13	29, Male	Ocular	12	II	I	I	40 mmHg	35 mmHg		No change	Infliximab, colchicine	Infliximab, colchicine
14	37, Female	Mucocutaneous and articular	14	II-V	I	I	40 mmHg			No change	Colchicine, BP	Colchicine
15	77, Female	Mucocutaneous and articular	35		II	I	40 mmHg			No change	Colchicine, BP	-
16	33, Male	Vascular	7	II	II	I	40 mmHg	20 mmHg	21.8	No change	Prednisolone, adalimumab	Adalimumab
17	52, Male	Vascular	16		I	I	40 mmHg	20 mmHg		No change	Prednisolone, colchicine, BP, azathioprine	BP, azathioprine

*Patients were ranked from highest to lowest according to initial sPAP values

**Classification of pulmonary hypertension at initial assessment. PH classification was based on probable causes of PH, in line with current guidelines, through a multidisciplinary evaluation

*BD* Behcet's disease, *PH* pulmonary hypertension, *TTE* transthoracic echocardiography, *NYHA FC* New York Heart Association Functional Capacity, *LVDD* left ventricular diastolic dysfunction, *sPAP* systolic pulmonary artery pressure, *MY* mitral regurgitation, *PAI* pulmonary artery involvement, *AR* aortic regurgitation, *GIS* gastrointestinal system involvement, *DM* diabetes mellitus, *HT* hypertension, *DVT* deep vein thrombosis, *CTEPH* chronic thromboembolic pulmonary hypertension, *COPD* chronic obstructive pulmonary disease, *CKD* chronic kidney disease, *CAD* coronary artery disease, *SVT* supraventricular tachycardia, *SLZ* sulfasalazine, *IFN* interferon-alpha, *BP* Benzathine benzylpenicillin, *MMF* mycophenolate mofetil

***”No change” indicates the absence of new cardiac symptoms, additional cardiovascular diagnoses, or treatment modifications

****Except for patient number three, none of the patients used PAH-specific medication

+ between the baseline and last evaluation this patient received Anti TNF alpha agent treatment for PAI

All patients with PAI and PH had previously been treated with high-dose steroids and/or immunosuppressive agents (primarily cyclophosphamide) and/or anti-TNF agents/interferon-alpha. None of the patients had active disease during the baseline or last evaluation period. Only one patient with Group IV PH was treated with PAH-specific agents. Data regarding treatments for Behçet's disease at baseline and during the last evaluation are presented in Table 1.

### Patients with group II and III PH

Eight patients previously diagnosed with Group II or III PH were re-evaluated using TTE. Five of these patients exhibited symptoms at the baseline assessment (FC ≥ 2). At the fifth-year evaluation, it was observed that only one patient remained symptomatic. In six patients, sPAP decreased to below 40 mmHg. In comparison, the sPAP remained ≥ 40 mmHg in two patients but did not show an increase relative to the baseline measurement (Table 1).

### Patients with group IV PH

All of the 4 patients with Group IV PH were evaluated at 5 year follow up visit. Two patients underwent pulmonary endarterectomy operation (PEA) during follow-up. In the first patient (Table 1, No: 2) PEA was performed after controlling pulmonary vasculitis with infliximab and corticosteroids. RHC findings before PEA were consistent with combined post and precapillary PH (mPAP; 35 mmHg, PVR;4 WU, PCWP; 25 mmHg). Due to residual defects in perfusion scintigraphy and persistent elevation of sPAP (60 mmHg) in TTE, RHC was performed 4 months later and revealed persistant combined post and precapillary PH (mPAP; 33 mmHg, PVR; 2.6WU, PCWP; 20 mmHg). Mitral valve replacement (MVR) was performed due to severe mitral insufficiency. RHC findings after MVR were as follows: mPAP; 24 mmHg, PVR; 1.7WU and PCWP; 17 mmHg. At 5 year follow up visit, sPAP on echocardiography was 45 mmHg and patients functional class was WHO-FC II. Second patient (Table 1 No:3) underwent PEA due to complete occlusion of the right main pulmonary artery and incomplete occlusion in the posterobasal segment of the lower lobe of the left lung complicated with precapillary PH (mPAP; 32 mmHg, PVR; 4.26 WU, PCWP; 15 mmHg). In control RHC performed six months after PEA, despite the observed improvement in hemodynamic parameters (mPAP; 28, PVR; 3.8, PCWP; 9), treatment with riociguat was commenced due to his persistent symptoms. Subsequent RHCs performed 6 months and 4 years after initiation of PAH-spesific therapy were still consistent with precapillary PH (mPAP; 24 and 30 mmHg, PVR; 3.6 and 3.3 WU, PCWP; 13 and 15 mmHg, respectively). At the 5-year follow-up, the sPAP on echocardiography was 40 mmHg and the patient's functional class was WHO-FC III. In other 2 patients, no deterioration was observed in functional capacity and sPAP was below 40 mmHg at 5 year follow up visit (Table 1).

### Patients whose PH group could not be determined

All 3 patients in this group were still asymptomatic at 5-years follow-up. One patient was evaluated with TEE and sPAP was < 40 mmHg (Table 1).

### Patients with PAI but without PH

Telephone contact was made with all 6 patients in this group. The WHO-FC class of 5 patients was classified as I, while one patient had a FC of 2. The latter patient had a TTE evaluation 5 years after the initial evaluation and sPAP was 30 mmHg. During the fifth-year thorax CT evaluation of this patient, findings included peripheral interstitial thickening, traction bronchiectasis, and ground glass appearance in the lower lobes.

## Discussion

In this study we presented data about the clinical follow-up of BD patients with PH after 5 years of PH diagnosis. All patients were alive at the end of the observation period. PH progression occurred only in patients with Group IV PH; two patients from this group underwent PEA operations. One patient with Group IV PH had mitral insufficiency operation due to persistent symptoms and post-capillary PH after PEA. PH class did not change in other patients.

Seyahi et al. investigated presence of PH (sPAP ≥ 35 mmHg by TTE) among patients with BD, systemic sclerosis and healthy controls [11]. They reported that the percentage of patients with PH were similar between BD and systemic sclerosis and both were higher than healthy controls. Prevalence of PH among BD patients in their study was 9% and only patients with vascular involvement had PH. However; they did not classify the causes of PH in their patients with BD and did not present data about follow-up of these patients. Recently, Manzari et al. reported that the mean sPAP of BD patients was higher than healthy controls (24.6 ± mmHg versus 22.81 ± mmHg, respectively) [14]. This was a cross-sectional study that lack data about the prevalence of PH and follow-up. In our previous study, conducted with 153 BD patients, PH prevalence was 11%, Group II and Group IV PH were the leading causes of PH [6].

In this study, severe and/or progressive PH was observed only in BD patients with PAI (Group IV PH). Presence of PH in BD patients with PAI at the time of diagnosis was found to be associated with mortality and was reported as a poor prognostic factor [7]. Moreover, these patients are at increased risk of progressive Group IV PH that can lead to right heart failure and death later in the disease course. PEA has been suggested to be an effective treatment option in this patient group [15, 16]. PEA was performed in 2 of 4 patients who had Group IV PH during follow-up period in our study. In other 2 patients sPAP was < 40 mmHg in their last evaluation and only one was mildly symptomatic. All the patients with PAI who did not have PH in their first evaluation were still asymptomatic after 5 years except one. However, sPAP was < 40 mmHg in this patient. Immunosuppressive treatment is highly effective in BD patients with PAI and most of the vascular lesions recover by time [17, 18]. Therefore, exact time to perform PEA operation is still unknown but it should be performed in BD patients who were on effectual immunosuppressive treatment and had well controlled disease activity with sequeal pulmonary vascular lesions [16]. Data about effectiveness of PEA in BD patients with chronic thromboembolic disease like conditions (symptomatic patient without PH) or balloon angioplasty in group IV PH are limited [5, 16]. Implementation of TTE for PH screening and monetarization in BD patients with PAI will provide valuable data for defining treatment strategy in this patient group.

Pulmonary arterial hypertension (PAH/Group I PH) is a rapidly progressive clinical condition with high mortality if left untreated [5]. Potentially; PAH associated with portal hypertension (Budd-Chiari syndrome) and/or drugs (cyclophosphamide, interferon-α) can occur in BD [5, 16]. Small vessel involvement and fistulas between systemic and pulmonary circulation are other conditions that may lead to PAH in these patient group [16]. Although we could not define the cause of PH in every BD patient in our study, all patients were alive after 5 years and overall in good condition which is unexpected in the presence of PAH. Therefore, PAH should be very rare in BD. There were no hint in the literature for the development of PAH associated with Budd-Chiari syndrome and/or interferon-α usage, in BD patients [6, 8]. On the other hand, physicians should always keep these conditions in mind while evaluating a BD patient with PH.

Left heart (Group II PH) and pulmonary diseases (Group III PH) are the most frequent causes of PH worldwide [5]. Coronary arterial involvement, valvular heart diseases, intra-cardiac thrombosis are among the manifestations of BD that can lead to Group II PH [19]. Cardiopulmonary co-morbidities of the patients can also contribute the development of PH. Cigarette smoking is quite common in BD patients due to its anti-inflammatory effects that decrease the occurrence of oral lesions which is the most common cause of COPD and the major risk factor for coronary heart disease [11, 20]. In our study, nearly all patients with Group II PH were even better on their visit after 5 years of PH diagnosis. They declared that they did not have additional cardiopulmonary diagnosis during this period. It’s really hard to define the exact cause of an organ dysfunction and/or PH in patients with systemic inflammatory diseases such as BD [10]. Therefore, besides controlling BD activity, adequate treatment of co-morbidities are mandatory in the follow-up.

Small number of patients and lack of hemodynamic data in most of the patients were the main limitations of our study. Although echocardiography is the primary method for screening PH, TTE has limitations, as it may result in both false positives and false negatives when compared to right heart catheterization (RHC) [21]. Even the impact of this limitation of TTE may increase by new definition of PH (mPAP > 20 mmHg). The lack of TTE data in patients who were only contacted through phone interviews and could not attend the in-person evaluation can also be considered another limitation of our study. On the other hand, relatively long time interval between two evaluations helped us to make decisions about PH course of the patients.

In conclusion; PH in BD can occur due to various causes and is not rare. The exact cause and the severity of PH should be determined in every patient. PAI in BD can lead to severe, progressive Group IV PH and these patients should be screened and carefully monitorized.

## Data Availability

The datasets generated and/or analyzed during the current study are available from thehttps://drive.google.com/drive/folders/16oypvnSnQfmbxDS5gN6_KxYiAVKB_Cfl?usp=sharing
